# Molecular characterization of the circadian clock in paediatric leukaemia patients: a prospective study protocol

**DOI:** 10.1186/s12887-023-03921-6

**Published:** 2023-03-04

**Authors:** Marius Ludwig, Alireza Basti, Müge Yalçin, Johannes H. Schulte, Angela Relógio

**Affiliations:** 1 Department of Pediatric Hematology and Oncology, Charité – Universitätsmedizin Berlin, Corporate Member of Freie Universität Berlin, Humboldt-Universität Zu Berlin and Berlin Institute of Health, 10117 Berlin, Germany; 2 Institute for Theoretical Biology (ITB), Charité—Universitätsmedizin Berlin, Corporate Member of Freie Universität Berlin, Humboldt-Universität Zu Berlin and Berlin Institute of Health, 10117 Berlin, Germany; 3 Molecular Cancer Research Center (MKFZ), Medical Department of Hematology, Oncology, and Tumour Immunology, Charité—Universitätsmedizin Berlin, Corporate Member of Freie Universität Berlin, Humboldt-Universität Zu Berlin and Berlin Institute of Health, 10117 Berlin, Germany; 4 Institute for Systems Medicine and Faculty of Human Medicine, MSH Medical School Hamburg, 20457 Hamburg, Germany; 5 German Cancer Consortium (DKTK), Partner Site Berlin, and German Cancer Research Center (DKFZ), 69120 Heidelberg, Germany; 6 Berlin Institute of Health, 10117 Berlin, Germany

**Keywords:** Paediatric leukaemia, ALL, AML, Circadian rhythms, Clock genes, Rhythmicity analysis, Circadian profiling

## Abstract

**Background:**

In many organisms, including humans, the timing of cellular processes is regulated by the circadian clock. At the molecular level the core-clock consists of transcriptional-translational-feedback loops including several genes such as *BMAL1*, *CLOCK*, *PERs* and *CRYs* generating circa 24-h rhythms in the expression of about 40% of our genes across all tissues. Previously these core-clock genes have been shown to be differentially expressed in various cancers. Albeit a significant effect in treatment optimization of chemotherapy timing in paediatric acute lymphoblastic leukaemia has previously been reported, the mechanistic role played by the molecular circadian clock in acute paediatric leukaemia remains elusive.

**Methods:**

To characterize the circadian clock, we will recruit patients with newly diagnosed leukaemia and collect time course saliva and blood samples, as well as a single bone marrow sample. From the blood and bone marrow samples nucleated cells will be isolated and further undergo separation into CD19^+^ and CD19^−^ cells. qPCR is performed on all samples targeting the core-clock genes including *BMAL1*, *CLOCK*, *PER2* and *CRY1*. Resulting data will be analysed for circadian rhythmicity using the RAIN algorithm and harmonic regression.

**Discussion:**

To the best of our knowledge this is the first study aiming to characterize the circadian clock in a cohort of paediatric patients with acute leukaemia. In the future we hope to contribute to uncovering further vulnerabilities of cancers associated with the molecular circadian clock and in particular adjust chemotherapy accordingly, leading to more targeted toxicity, and hence decreased systemic toxicities.

## Background

In many organisms, including humans, the timing of cellular and physiological processes is regulated by an internal time generating machinery—the circadian system. In mammals, the circadian clock comprises a central pacemaker located in the suprachiasmatic nucleus in the brain, which is entrained to the geophysical time via (mostly) light signals and in turn synchronizes peripheral clocks in every cell. Cellular clocks are formed by molecular networks of interconnected and autoregulatory transcriptional-translational feedback loops, which generate 24-h rhythms in gene and protein expression and thus account for the proper timing of cellular processes [1]. Circadian oscillations are found in about 40% of all genes. Such clock-controlled genes [2, 3] are involved in many important processes including metabolism, sleep–wake rhythm, memory formation, and immunity [4]. Circadian regulation ensures for organismal adaptation of not only cellular processes, but also physiology and behaviour to the time-varying demands of the day-night cycles [5, 6]. Dysregulation of the circadian clock is associated with pathological phenotypes including sleep and mental disorders [7–11], obesity [12], neurodegeneration [13, 14] and cancer [15, 16].

In particular, in pathologies where the treatment can cause severe side effects in the patients, like in cancer, promising studies have reported a variation in toxic effect of anti-cancer drugs depending on the time of treatment [17–19]. On the other hand, the treatment of the above-mentioned pathologies can further disrupt the clock and contribute to some of the observed side effects of anticancer drugs.

Thus, to investigate the circadian clock in a pathological context, particularly in cancer is timely and of great medical relevance.

Acute leukaemia (AL) is the most common form of paediatric cancer and account for approximately 30% of childhood malignancies [20]. Previously published studies reported a leukaemia specific dependency on *Bmal1* and *Clock* in a murine model of acute myeloid leukaemia (AML) [21]. Furthermore, *Bmal1* and *Per2* promotors were found to be hypermethylated in RPMI8402, a human T-cell lymphoblastic leukaemia cell line [22]. Even though a significant effect of chemotherapy timing has been reported in acute lymphoblastic leukaemia (ALL) therapy [23] the mechanistic role of the molecular circadian clock in ALL remains elusive.

The planned study aims at profiling the circadian clock, via characterizing the expression of circadian clock genes (*BMAL1* and *PER2*) in healthy children, and children suffering from ALL and AML, and to correlate it with the age group, the presence of leukaemia and the administration of anti-leukaemia therapy. In particular, it enables to investigate the expression of the circadian clock in leukaemia cells at diagnosis and under therapy, and to compare it with the circadian rhythm of healthy cells in the organism of the same patient. The expression of clock genes can be measured from biological samples like blood or saliva, and RT-qPCR assays have been established in our group for assessment of circadian gene expression levels in human samples. Preclinically, we have shown that circadian rhythms are detectable also in malignant cells, though with different degrees of dysregulation, which can influence therapy response and tumour progression in colorectal carcinoma [15, 24, 25].

In the future, the knowledge acquired through this study may potentially be used to adapt leukaemia therapies to the circadian rhythm of the patient to reduce treatment toxicity and resulting side effects leading to an overall optimization of treatment efficacy.

## Methods/ design

### Objectives

The essential main hypotheses, which will be investigated equally, are:

- 1. a circadian rhythm is present in children at the level of the whole organism and may be dependent on age group and other covariates, such as the presence of leukaemia.
- 2. leukaemia treatment affects the circadian rhythm in children at the level of the whole organism.
- 3. the circadian rhythm in the leukaemia cells may differ from the circadian rhythm from healthy cells in the same patient; the onset of treatment affects the circadian rhythm of the whole organism and the leukaemia cells.

### Design

**Fig. 1:**
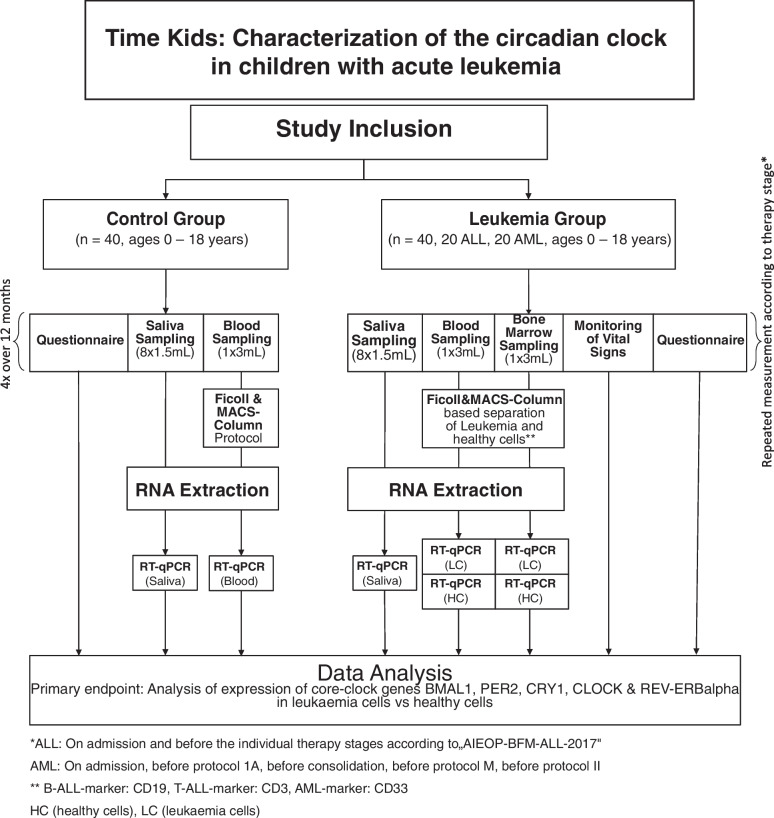
Study protocol flow chart. After meeting the study inclusion criteria, patients and control participants are included in their respective groups. Saliva, blood and bone marrow sampling is carried out according to the scheme in Fig. 2. From the blood and bone marrow samples PBMC are isolated and further undergo separation into CD19^±^ cells for ALL and CD33^±^ for AML. Afterwards RNA is extracted, qPCR analysis is carried out to quantify the expression of core-clock genes. This procedure is repeated for each patient

This is a single-centre follow-up observational study of children with ALL and AML diagnosed at the Department of Paediatric Haematology, Oncology and Stem-cell Transplantation at the Charité University Hospital, Berlin during the time of 2021–2023. This study aims to characterize the overall circadian clock of children with AL, as well as the circadian clock of leukaemia cells in a cohort of patients (*n* = 40) and controls (*n* = 40) (Fig. 1).

### Inclusion and exclusion criteria

**Fig. 2:**
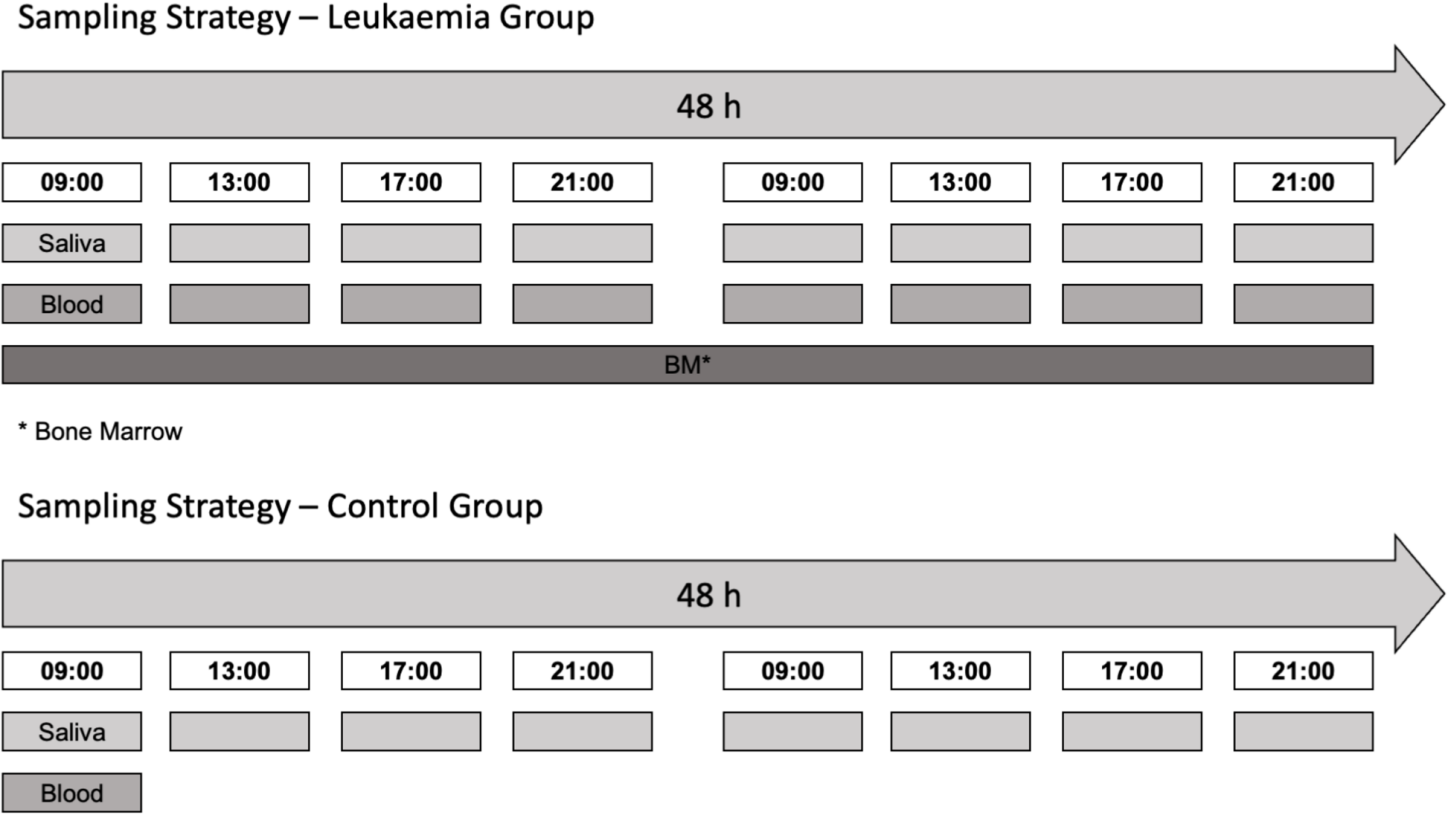
Sampling scheme for leukaemia patients and control probands. Saliva sampling will take place over the time of two days with sampling time points being 09:00, 13:00, 17:00 and 21:00 on two consecutive days. Blood will be sampled following the same schedule in leukaemia patients. In the control group, blood sampling will take place once. Bone marrow sampling will occur only in leukaemia patients during the first 48 h after admission in the hospital

Participants of the study are to be recruited from patients admitted to the Department of Paediatric Oncology, Haematology and Stem-cell Transplantation of the Charité Medical University, Berlin. Participation in the study is voluntary. Included into our control group are any healthy subject being evaluated as potential donors for bone marrow transplantation. Patients with an urgent suspicion of or secured new diagnosis of AL, in whom the start of treatment is not expected for > 24 h due to incomplete baseline investigations are included into our cohort of leukaemia patients. For all participants, inclusion requires a sufficient ability of the subject and family to cooperate so that saliva collection (Fig. 2) does not involve coercion or suffering for the subject and an agreement to participate in the study.

Exclusion criteria include a lack of consent to participate in the study, which can be withheld at any time without providing a reason. Furthermore, patients with AL and a bodyweight less than 3 kg are excluded due to low blood volume.

### Sample size

**Fig. 3:**
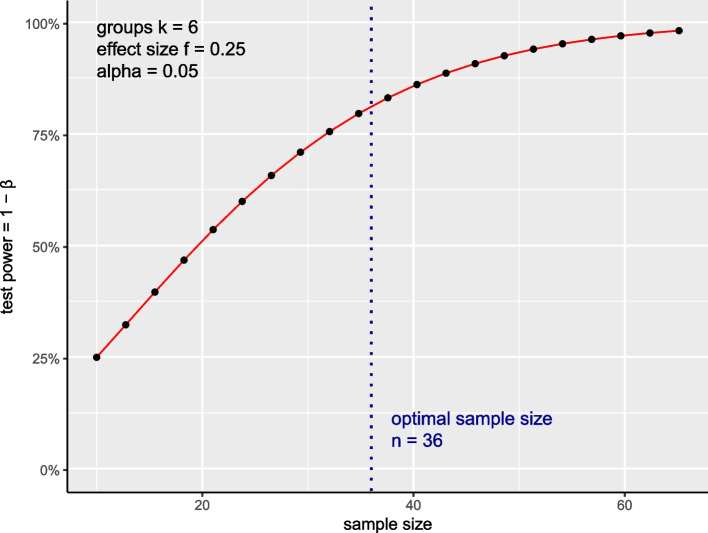
Graphical illustration of optimal sample size calculation for the study protocol. The plot illustrates the range of sample size values and corresponding test power. The power anova test setting was used to compute the sample size (α (significance) = 0.05; statistical power = 0.8; k_*numberofgroups*_ = 6; f (effect size) = 0.25). Dashed blue line indicates the optimal sample size (n) that needs to be collected for the detection of significant results according to pre-determined thresholds in each experimental group

To calculate the sample size, we carried out a power analysis, which allows calculation of sample sizes that enable obtaining significant and realistic results based on the experimental design. To estimate the optimal sample size for the groups in our study, we used”pwr.anova.test” function from “pwr” package as implemented in R. Due the lack of prior knowledge regarding the mean and variance for this particular experimental setup, we carried out a calculation using a moderate effect size (f) as previously suggested by Bausell and Li [26]. In addition, to compute the sample size we set up a threshold for significance α (also known as security) = 0.05, statistical power = 0.8, and k = 6 (2 × measurement for case group and 4 × measurement for control group), which represent number of groups in our study design. Using these criteria, our analysis suggested an optimal sample size of *n* = 36 per experimental group to detect significant changes in our study (Fig. 3).

### Data collection

Biological Sample Collection: For saliva collection, 1.5 mL unstimulated saliva is collected in tubes containing 1.5 mL RNAprotect Tissue Reagent (Qiagen, Hilden, Germany), mixed immediately and stored at -20 °C until further analysis. Saliva will be collected at four time-points per day during two consecutive days. RNA extraction and gene expression analysis from saliva samples will be performed by Qiagen GmbH laboratory (Qiagen, Hilden, Germany). Blood samples will be collected once from the healthy control participants and at four time-points over two consecutive days (eight samples in total) from patients, with 3 mL blood drawing per sampling. To separate leukaemic and normal cells we will firstly isolate the mononuclear cells from blood/bone marrow samples using a ficoll protocol. Mononucleated cells will be separated via a density gradient using PMBC 24+ Spin Medium (pluriSelect). The mononuclear cells will be incubated with antibody-conjugated magnetic microbeads (Miltenyi Biotec, Bergisch Gladbach, Germany) specific for CD19 (B-cell-ALL), CD3 (T-cell-ALL) or CD33 (AML) an dpassed through MACS-column (Miltenyi Biotec, Bergisch Gladbach, Germany) exposed to a magnetic field. Target cells will be trapped in the column and washed out when the column is removed from the magnetic field [27]. Healthy and cancer cell pellets will be frozen or directly lysed with RLT lysis buffer (Qiagen, Hilden, Germany) and stored at -20 °C until further analysis. RNA extraction from blood cells will be performed using RNeasy Micro Kit (Qiagen, Hilden, Germany) with the optional gDNA removal step using DNase. Extracted total RNA will be reverse transcribed into cDNA with M-MLV reverse transcriptase (Invitrogen, Thermo Fisher Scientific, Carlsbad, CA, USA), random hexamers (Thermo Fisher Scientific, Waltham, MA, USA) and dNTPs Mix (Thermo Fisher Scientific, Waltham, MA, USA). qPCR will be performed using human Qiagen Primer assays (Qiagen, Hilden, Germany) and SsoAdvanced Universal SYBR Green Supermix (Bio-Rad laboratories, Hercules, CA, USA) in 96-well plates.

The analysis of the circadian rhythm of patients, based on saliva samples will be carried out as described above, before, during and after treatment.

In particular, for patients with ALL: on admission a complete course of sampling is scheduled including sequential (time course) blood and saliva sampling, as well as a single blood marrow sample. Sequential saliva sampling will occur on follow-up timepoints before and after the individual therapy phases according to the protocol "AIEOP BFM-ALL 2017".

Example Medium Risk ALL: 1. Sequential blood, sequential saliva sampling and a single bone marrow sample on admission; 2. Sequential saliva sampling before and after protocol 1A; 3. Sequential saliva sampling before and after consolidation; 4. Sequential saliva sampling before and after protocol M; 5. before and after protocol II. For patients with acute myeloid leukaemia (AML): Sequential blood sampling, sequential saliva sampling and a single bone marrow sample are scheduled on admission, sequential saliva samples will be collected before and after the individual therapy blocks according to the current protocol of the AIEOP study group.

### Measurements

Experimental part: core-clock gene expression will be measured in healthy and cancer patients using multiple peripheral biological samples over time (saliva, blood and bone marrow samples), as depicted in Fig. 1 and Fig. 2. Expression from following core-clock genes will be measured: *BMAL1*, *PER2*, *CRY1*, *CLOCK* and *REV-ERBalpha*. Saliva will be collected at eight time-points over two days, with 1.5 mL saliva per time-point and used for subsequent RNA extraction and qPCR analysis of core-clock genes. In case of RNA left-over from saliva samples after qPCR analysis, the NanoString technology can be used to measure the expression of a broader range of core-clock and clock related genes. Peripheral blood samples will be collected at eight time-points over two consecutive days from cancer patients and once from healthy participants, with 3 mL blood per time-point (Fig. 1–2). Peripheral Blood Mononuclear Cells (PBMCs) from whole blood will be separated using the Ficoll method and will be used for MACS-column based separation of healthy and cancer cells using B-ALL (CD19), T-ALL (CD3) or AML (CD33) specific markers. Subsequently, RNA will be extracted from the cells after separation and used for qPCR assay to detect core-clock gene expression. Bone marrow samples will be collected once from cancer patients with 3 mL sample per time-point. Samples will undergo Ficoll separation and MACS-column based separation of healthy and cancer cells similar to blood samples, and used for RNA extraction and qPCR for measuring core-clock gene expression (Fig. 1–2).

### Statistical analysis

#### Assessment of circadian rhythm profile of probands

The expression values of the individual clock genes will be measured by RT-qPCR and subsequently will be evaluated using the ddCT method.[28] Each gene expression value will be normalized to the mean value of all time points and to the expression of housekeeping gene (*GAPDH*). The temporal expression profile of each short-listed clock gene is then tested individually for circadian rhythmicity using the RAIN algorithm [29]. Harmonic regression will be used in the analysis of circadian data [30, 31]. For this, the model formulated as: y(t) = m + acos(ωt) + bsin(ωt) is fitted to the measured data to determine the absolute (A = √(a2 + b2)) and relative amplitude (compared to the mean), as well as the phase (tan φ = b/a), the p-value and the confidence interval. For this purpose, and harmonic regression method as implemented in the R package HarmonicRegression [32] will be used.

#### Differential rhythmicity and expression analysis

For genes with significant circadian expression profiles in saliva samples collected from different individuals, the circadian properties can be compared. These circadian changes may include alterations in oscillatory properties such as absolute amplitude (the difference between maximum and minimum gene expression values); phases (the time point of maximum gene expression) and period (the time difference between two repetitive cycles; ~ 24 h). The differences are considered significant if the acrophase difference of a gene between samples is ≥ 4 h. In addition, differences in the amplitudes can also be compared among different test persons. The DODR (Detection of Differential Rhythmicity) method will be used to detect significant differential changes for pairwise comparisons between each test person [33]. DODR p-values will be adjusted using Benjamini-Hochberg procedure, and a significance threshold will be set for *q* < 0.05 for significance changes. Following the linear modelling including the circadian harmonics, the coefficient representing the mean gene expression level will be used to compare mean gene expression changes between each participant data as implemented in R limma package [34].

#### Statistical analysis for primary and secondary outcomes of the study

The primary objective and consideration of success of this study is dependent on detection of circadian profiles from collected saliva and blood samples of paediatric leukaemia patients. If the circadian profiles can be detected in clock genes from different samples, the study will be considered successful. Secondary outcome variables include impact of leukaemia treatment on circadian rhythms, which might be further changed by covariates, such as the presence of leukaemia, age or leukaemia treatment. Descriptive statistics will be evaluated for demographic information and clinical variables and the distribution of the data will be assessed. Subsequently, one-way ANOVA with Tukey post-hoc test, in case of parametric test assumptions are met or for non-parametric dataset Kruskal–Wallis test will be used to compare differences between paediatric leukaemia patients and respective controls. All statistical tests will be evaluated at a statistical significance threshold set to (α) 0.05. Of note, additional parameters can be considered for stratification and potential cofounding effects for the analysis, such as disease stage, which may influence the analysis and results. For this purpose, ANCOVA (The Analysis of Covariance), which consist of a combination of ANOVA and linear regression will be used to control for the effect of potential confounding variables in this study, and to avoid their influence on the outcome. All statistical analyses will be carried out with R statistical programming software.

## Discussion

AL is the most common malignancy in childhood amounting to approximately 30% of all paediatric malignancies [20]. Children with AL typically present symptoms associated with bone marrow insufficiency such as fever, paleness, fatigue and petechial bleeding. Further symptoms include hepatosplenomegaly, lymphadenopathy, and musculoskeletal pain. To confirm the diagnosis of AL, peripheral blood and bone marrow samples are required to conduct cytology, cytochemistry, immunophenotyping, cytogenetics and molecular genetic testing [35]. The 5-year event-free survival of paediatric patients is 80–90% [36]. Once the diagnosis is confirmed risk stratified treatment is initiated. Treatment options include conventional chemotherapy, stem-cell transplantation, as well as immunotherapy [37]. The most severe side effects include tumorlysis-syndrome, infection, bleeding as well as HPA (Hypothalamic–Pituitary–Adrenal) axis suppression [38].

To the best of our knowledge this is the first study analysing the circadian clock in children newly diagnosed with leukaemia.

Chronomodulated treatment in cancer takes advantage of the circadian rhythms in cancer and/or host cells. In fact, cancer cells have been reported to have a variety of circadian profiles, depending on the type and progression stage of a given cancer, which range from arrhythmic [39], rhythmic and in phase with healthy cells [40] or rhythmic and out of phase with healthy cells [19]. For best chronotherapy outcome, the toxicity susceptibility pattern of cancer cells should be ideally antiphase to those of healthy cells, and thus an optimal treatment timing could be set at a time point of least toxicity for healthy cells. Several animal studies [41–43] and clinical trials with chronomodulated therapy regimen for nasopharyngeal cancer [44, 45], colorectal cancer [46], endometrial [47] and ovarian cancer [48] have shown reduced toxic side effects and improved efficacy against conventional therapy. In a recent systematic review including 18 clinical studies with 2547 patients, the authors concluded that chronomodulated chemotherapy results in the reduction of toxicity while maintaining treatment efficacy in most cases (61% of the studies) [18]. However, to our knowledge, no study has reported treatment effects on the patients’ circadian clock in healthy and leukaemia cells in ALL.

A few limitations are associated with this study including its nature as a single centre study and limited sample size, logistical challenges generated by the unforeseeable arrival of newly diagnosed patients with AL, as well as hindered collection of saliva samples, especially in very young children. Nevertheless, with this study we expect to obtain valuable inputs leading to a better understanding and potential treatment optimization of ALL, including to characterize for the first time the circadian rhythm of children with AL using sequential sampling on a systemic, as well as cellular level. To reduce bias due to the known seasonal changes in the expression of circadian clock genes [8], we aim for a continuous recruitment of subjects over the entire study-period. Furthermore, to allow for an intra-individual comparison of the clock profile between disease vs heathy cells, we will collect both leukaemic and normal cells from the same patient, at the very same time. Moreover, magnetic microbead-based cell selection has been previously established to isolate leukaemia cells, and the possibility of contamination during cell separation is negligible [49]. To quantify the specificity of the leukaemia specific markers, the percentage of healthy progenitor cells carrying this marker will be verified via the immunophenotyping included in the standard diagnostic procedure. To increase the sensitivity of the cell separation process an increased microbead concentration will be used to minimize false negatives in the flow through, as previously demonstrated [50]. Importantly, magnetic microbead-based cell selection will allow for a swift processing of newly collected samples. A deeper understanding of the mechanisms involved in potential alterations in the circadian clock of AL might contribute to a better understanding of leukaemia specific vulnerabilities and hence potentially ameliorate the efficacy of therapy and mitigate unwanted side effects and complications. Furthermore, the characterisation of the circadian clock in leukaemia cells might further contribute to a deeper understanding of clock-related mechanisms in cancer on a more general level, potentially paving the way for the implementation of chronomodulated therapy strategies or clock specific cancer therapies in a clinical setting.

## Data Availability

The datasets that will be generated and analysed during this current study are not yet available because the study is presently ongoing. We will obtain informed consent for publication of the dataset from participants at the point of recruitment to the study. If patients do not wish their data to be shared and used they will inform the study doctor. Patients can also withdraw at any time from the study without providing a reason. Datasets described in the manuscript, including all relevant raw data, will be available from the corresponding author upon reasonable request once recruitment and data collection is complete.

## Abbreviations

AL: Acute Leukaemia
ALL: Acute Lymphoblastic Leukaemia
AML: Acute Myeloid Leukaemia
ANCOVA: The Analysis of Covariance
DODR: Detection of Differential Rhythmicity
HPA: Hypothalamic–Pituitary–Adrenal
PBMC: Peripheral Blood Mononuclear Cell
